# Estimated Cost-effectiveness of Genetic Testing in Siblings of Newborns With Cancer Susceptibility Gene Variants

**DOI:** 10.1001/jamanetworkopen.2021.29742

**Published:** 2021-10-18

**Authors:** Grace O’Brien, Kurt D. Christensen, Haley K. Sullivan, Natasha K. Stout, Lisa Diller, Jennifer M. Yeh, Ann Chen Wu

**Affiliations:** 1 Boston Children's Hospital, Boston, Massachusetts; 2 Harvard Pilgrim Health Care Institute, Boston, Massachusetts; 3 Harvard Medical School, Boston, Massachusetts; 4 Harvard University, Cambridge, Massachusetts; 5 Dana-Farber Cancer Institute, Boston, Massachusetts

## Abstract

This economic evaluation examines the costs and benefits of cascade testing of siblings of newborns with cancer susceptibility gene variants.

## Introduction

Newborn population-based genetic screening may reduce pediatric cancer deaths and could be cost-effective.^1^ Testing siblings of newborns with variants (ie, cascade testing) could further improve population health.^2^ Economic studies of cascade testing have focused on adults, although evidence suggests that cost-effectiveness improves for some cancer syndromes when surveillance is initiated in younger individuals.^3^ Our objective was to estimate the benefits and costs of cascade testing of siblings of newborns with cancer susceptibility gene variants.

## Methods

This economic evaluation study followed the Consolidated Health Economic Evaluation Reporting Standards (CHEERS) reporting guideline. This study was deemed exempt from review, and informed consent was not required because the institutional review board at Harvard Pilgrim Health Care determined the project did not meet the definition of human participant research.

Using the Precision Medicine Policy and Treatment (PreEMPT) model^1^ created in 2020, we estimated outcomes in siblings of newborns who were born with pathogenic or likely pathogenic variants in 1 of 11 pediatric cancer genes (ie, *RET*, *RB1, TP53, DICER1, SUFU, PTCH1, SMARCB1, WT1, APC, ALK*, or *PHOX2B*). Genes were selected based on associations with increased risk of very early onset malignant neoplasm and available surveillance guidelines. Children with variants were assumed to have 1 newborn sibling, based on US Census Bureau data. In the base case, we assumed de novo variants were rare, and siblings had 50% likelihoods of having the same germline variants as probands. Because of the variation and uncertainty in de novo variant rates by gene,^4^ we then varied assumptions about having the same germline variants to 5% (ie, very high de novo rate) and 25% (ie, moderate rate) in scenario analyses. Model outcomes included lifetime cancer deaths averted and cost-effectiveness relative to no cascade testing, calculated from a societal perspective with 3% discounting for costs and life-years (LYs). Cascade testing costs included Sanger sequencing (ie, $200) and clinical visits (ie, $188) before initiating surveillance. Surveillance costs were based on 2018 Medicare reimbursement rates. Sensitivity analyses varied costs of sequencing, clinical visits, and surveillance by 25%. To capture uncertainty, we conducted 1000 simulations where we sampled model parameters from their underlying distributions. We report means and 95% uncertainty intervals (UIs), defined as the 2.5 and 97.5 percentiles of estimates from 1000 simulations. Analyses were conducted in 2021 using R version 4.1.0 (R Project for Statistical Computing). Statistical tests were not conducted, given estimates were derived from simulation modeling rather that population sampling.

## Results

In a cohort of 3.7 million newborns, the model estimated 1584 newborns (95% UI, 1230-2026) and 792 siblings (95% UI, 615-1013) would carry variants. An estimated 116 siblings (95% UI, 98-139) carrying variants would develop cancer before age 20 years. If these siblings underwent surveillance, 15 cancer deaths (95% UI, 11-20) would be averted, representing a 52% reduction (95% UI, 45%-59%) (Table 1). Compared with usual care, sibling cascade testing had an incremental cost-effectiveness ratio (ICER) of $16 910 per LY gained (95% UI, $7060-$31 440). ICERs for individual genes ranged from cost-saving (eg, *WT1*, *SUFU*) to $52 100 per LY gained (*TP53*), and scenario analyses on the frequency of de novo variants did not substantially change ICER estimates (Table 2). ICERs were robust in sensitivity analyses on sequencing and initial visit costs ($16 650-$17 160 per LY gained) and surveillance costs ($10 040-$24 380 per LY gained).

**Table 1.** Modeled Outcomes of Newborn Genetic Screening and Sibling Cascade Testing for a Simulated Cohort of 3.7 Million Newborns: Base Case

Outcome	Screening	
	11-gene panel, No. (95% UI)	Sibling cascade (95% UI)
Eligible for screening	3 700 000	1584 (1230-2026)
P/LP variant carriers	1584 (1230-2026)	792 (615-1013)
Cancer cases^a^	1803 (1756-1850)	116 (98, 139)
Cancer death		
Under usual care	406 (383-429)	29 (23-36)
Averted with genetic screening	32 (24-42)	15 (11-20)
Averted with genetic screening, %	8 (6-10)	52 (45-59)
Incremental cost-effectiveness ratio, ($ per LY gained)^b^	244 860 (181 840-327 510)	16 910 (7060-31 440)

Abbreviations: LYs, life-years; P/LP, pathogenic or likely pathogenic; UI, uncertainty intervals.

^a^ Cancer cases for the 11-gene panel include all medullary thyroid carcinoma, retinoblastoma, adrenocortical carcinoma, choroid plexus, rhabdomyosarcoma, osteosarcoma, rhabdoid tumors, pleuropulmonary blastoma, medulloblastoma, neuroblastoma, Wilms tumor, and hepatoblastoma, including those not attributable to 1 of the 11 genes. Siblings were only at risk for developing the cancer associated with the gene for which they were assumed to undergo genetic screening.

^b^ Calculated as the ratio of expected incremental costs divided by the expected incremental life years among 1000 simulations.

**Table 2.** Cost-effectiveness of Sibling Cascade Testing Compared With Usual Care

Sibling subgroup	Base case^a^				Scenario analyses, $	
	Mean (95% UI)			ICER (95% UI), $^d^	ICER (95% UI) for moderate rate of de novo variant^d^^,^^e^	ICER (95% UI) for a very high de novo variant rate^d^^,^^f^
	Siblings eligible for cascade testing, No.^b^	Incremental costs per 100 siblings, $^b^	Incremental life-years per 100 siblings^c^			
All combined	1584 (1230 to 2026)	481 660 (261 750 to 662 800)	28 (20 to 40)	16 910 (7060 to 31 440)	17 610 (7610 to 32 360)	23 220 (11 760 to 40 150)
*ALK*	28 (6 to 83)	−807 190 (−2 597 620 to 548 260)	132 (14 to 283)	Cost-saving (cost-saving to 37 750)	Cost-saving (cost-saving to 39 160)	Cost-saving (cost-saving to 50 370)
*APC*	361 (195 to 576)	173 350 (101 820 to 217 130)	5 (1 to 11)	39 020 (9450 to 183 370)	43 440 (11 270 to 200 280)	78 780 (25 930 to 335 590)
*DICER1*	94 (24 to 209)	27 310 (−850 170 to 331 910)	31 (7 to 99)	5730 (cost-saving to 46 550)	6600 (cost-saving to 49 330)	13 570 (cost-saving to 71 590)
*PHOX2B*	14 (0 to 72)	−812 180 (−4 081 340 to 658 350)^g^	132 (3 to 398)^g^	Cost-saving (cost-saving to 59 620)^g^	Cost-saving (cost-saving to 61 950)^g^	Cost-saving (cost-saving to 80 600)^g^
*PTCH1*	32 (15 to 83)	−1 036 670 (−2 235 660 to −15 520)	115 (44 to 201)	Cost-saving (cost-saving to cost-saving)	Cost-saving (cost-saving to 100)	Cost-saving (cost-saving to 3780)
*RB1*	97 (65 to 183)	59 930 (−182 550 to 313 210)	37 (17 to 58)	2640 (cost-saving to 18 150)	3210 (cost-saving to 19 310)	7810 (cost-saving to 28 530)
*RET*	39 (9 to 122)	−2 095 600 (−6 956 150 to 410 930)	51 (7 to 153)	Cost-saving (cost-saving to 56 050)	Cost-saving (cost-saving to 58 400)	Cost-saving (cost-saving to 80 600)
*SMARCB1*	38 (20 to 93)	−2 236 550 (−4 360 500 to −17 750)	230 (84 to 380)	Cost-saving (cost-saving to cost-saving)	Cost-saving (cost-saving to 30)	Cost-saving (cost-saving to 1840)
*SUFU*	32 (15 to 85)	−1 049 030 (−2 333 510 to 17 430)	116 (42 to 210)	Cost-saving (cost-saving to 390)	Cost-saving (cost-saving to 830)	Cost-saving (cost-saving to 4440)
*TP53*	827 (558 to 1128)	1 044 250 (798 490 to 1 208 130)	21 (10 to 37)	52 100 (21 710 to 120 860)	53 090 (22 250 to 122 860)	61 000 (26 600 to 138 880)
*WT1*	22 (5 to 73)	−196 180 (−889 130 to 206 040)	43 (5 to 103)	Cost-saving (cost-saving to 37 750)	Cost-saving (cost-saving to 41 530)	3990 (cost-saving to 71 230)

Abbreviations: ICER, incremental cost-effectiveness ratio; UI, uncertainty intervals.

^a^ Assumes a 50% likelihood siblings carry the same variant as probands.

^b^ Number of siblings based on variant carriers identified via newborn screening of a birth cohort of 3.7 million individuals with a 11-gene panel.

^c^ Sibling cascade testing compared with usual care.

^d^ Calculated as the ratio of expected incremental costs divided by the expected incremental life-years among 1000 simulations. 95% UI is based on ICERs calculated for each simulation. ICERs were defined as cost-saving if sibling cascade testing had higher incremental LYs and lower incremental costs compared with usual care.

^e^ Assumes a 25% likelihood siblings carry the same variant as probands.

^f^ Assumes a 5% likelihood siblings carry the same variants as probands.

^g^ Excludes 4 simulations (out of 1000) which included no cases of *PHOX2B*.

## Discussion

In this study, we estimated that sibling cascade testing would identify approximately 800 siblings with variants, avert half of projected cancer deaths before age 20 years among these individuals, and would have high value.^5^ Results align with adult studies that find relatives’ genetic testing cost-effective compared with standard screening.^2^

Limitations to our work included consideration of only 11 genes, exclusion of costs and benefits of parental cascade testing, entry of siblings into the model at birth, and omission of impact on adult-onset cancer outcomes. Benefits assumed full adherence to surveillance and previously used assumptions regarding early detection resulting in improved outcomes.^1^ We did not consider how siblings of variant carriers who developed cancer may already undergo surveillance or genetic testing. Notably, the most cancer deaths averted (ie, 80%-89%) accrued among siblings of healthy newborns. Findings demonstrate how sibling cascade testing would enhance newborn screening efforts and how targeted screening approaches may be more efficient than universal screening to achieve population-level benefits.^6^
